# Defining the mechanisms underlying cyclin dependent kinase control of HIF-1α

**DOI:** 10.18632/oncotarget.28208

**Published:** 2022-03-03

**Authors:** Noel A. Warfel

**Affiliations:** ^1^University of Arizona Cancer Center, Tucson, AZ, USA; ^2^Department of Cellular and Molecular Medicine, University of Arizona, Tucson, AZ, USA

**Keywords:** hypoxia, HIF-1, cyclin dependent kinase, SMURF2

***Commentary on:** Zhao and El-Deiry. Identification of Smurf2 as a HIF-1α degrading E3 ubiquitin ligase. Oncotarget. 2021; 12:1970–79. https://doi.org/10.18632/oncotarget.28081. [PubMed]*

Constitutive activation of HIF-1α is common in human cancers, regardless of oxygen tension. While the majority of HIF-1 activation can be attributed to lack of oxygen in the tumor microenvironment, numerous non-hypoxic stimuli have also been shown to regulate HIF-1α levels. Stabilization of HIF-1α in normoxia has been attributed to genetic alterations, most notably loss of the von Hippel-Lindau (VHL) tumor suppressor gene, the primary E3 ligase responsible for targeting HIF-1α for proteasomal degradation [1]. In recent years, multiple new proteins and post-translational modifications have been implicated in the oxygen-independent control of HIF-1α. This includes alternative E3-ubiquitin ligases that target HIF-1α for proteasomal degradation (RACK, CHIP, HAF, etc.) [2], as well as binding proteins that enhance stability, such as HSP90 [3]. Another critical event that impacts HIF-1α levels and activation is phosphorylation. Numerous phosphorylation sites and upstream kinases, including PKA and PIM1 kinases, have been identified and shown to modulate HIF-1α protein stability in both normoxia and hypoxia [4–6]. Regardless of the mechanism, stabilization of HIF-1α in normoxia results in the constitutive upregulation of genes that initiate and sustain signaling pathways that drive cellular processes that support tumor growth and metastasis. As a result, identifying new mechanisms regulating HIF-1 is crucial to our understanding of cancer progression and developing more effective therapies.

Prior research from the El-Deiry lab was the first to demonstrate that the cyclin dependent kinases CDK1 and CDK4/6 are sufficient to stabilize HIF-1α, independent of hypoxia or VHL. Following up on these exciting findings, Zhou and El-Deiry utilized an unbiased proteomic screen to identify SMAD specific E3-ubiquitin protein ligase 2 (SMURF2) as a novel E3 ligase controlling HIF-1α levels downstream of CDK4/6, regardless of oxygen tension. Moreover, mass spectrometry analysis revealed loss of phosphorylation of HIF-1α at Ser451 in cells treated with palbociclib, raising the possibility that this site could be important for maintaining HIF-1 stability. Interestingly, recent work from our group showed that phosphorylation of HIF-1α at Thr455 by PIM1 blocks HIF-1α degradation by disrupting prolyl hydroxylase domain (PHD) protein binding and hydroxylation, which is the initiating step in the canonical HIF-1α degradation pathway [4]. While the mechanism appears to be distinct, since PIM1 blocks VHL-mediated degradation, the close proximity of these sites and their localization within the oxygen dependent degradation domain in HIF-1α points to the importance of post-translational modifications to this region for the regulation of HIF-1α protein stability through both canonical and non-canonical means. Importantly, analysis of the TCGA data showed that high levels of SMURF2 correlated with significantly better overall survival and disease-free survival in clear cell renal cancer, in which over 80% of patients lack functional VHL and display high basal levels of HIF-1α. In a parallel study, the same authors leveraged their findings to test whether targeting multiple molecules that stabilize HIF-1α simultaneously enhanced therapeutic response. Strikingly, the combination of FDA-approved CDK4/6 inhibitors and HSP90 inhibitors showed enhanced inhibition of HIF-1 activity and synergistic anti-tumor effects in models of renal and colon cancer lacking VHL and Rb [7]. Taken together, these studies describe a new mechanism responsible for the activation of HIF-1 in human cancer and provide a strong rationale for the use of CDK4/6 inhibitors to target HIF-1, particularly in tumors lacking VHL or harboring other signaling alterations that promote the constitutive activation of HIF-1.
